# P090: Importance of the information system in the fight against multi-drug resistant bacteria

**DOI:** 10.1186/2047-2994-2-S1-P90

**Published:** 2013-06-20

**Authors:** S Izoard, A Brenet, P Astagneau, Z Kadi

**Affiliations:** 1 Picardie regional center for nosocomial infections control, CCLIN Paris Nord, Amiens, France; 2 CCLIN Paris Nord, Paris, France

## Introduction

The Picardie region is one of the regions in which the incidence of methicillin-resistant Staphylococcus aureus (MRSA) is high (0.4 / 1000 Hospitalization Days in Picardie in 2011) compared to the French national average (0.7 / 1000 Hospitalization Days in 2011).

## Objectives

That is why the regional center for nosocomial infections control in 2012 made an assessment study on the actions and the resources involved in prevention and control of MRSA and Multi-Drug Resistant Bacteria (MDRB).

## Methods

The survey was proposed to 82 hospitals in the region using on-line or paper questionnaires forms. Data were entered and analyzed using Epi-Info 6.04d software.

## Results

Overall 41 hospitals (50% of the hospitals in the region) participated in this investigation. 66% of them reported having a system of continuous monitoring of MDRB for the identification of clustered cases and 39% of the hospitals reported having a similar device for the identification of re-hospitalized cases. In addition, 63% of respondents indicated that they did not develop transmission means for the patients with MDRB to inform the hosting facility during a subsequent hospitalization. When a case of patients with MDRB occurs in a hospital, 88% of them track cases of re-hospitalized patients thanks to patient records and not via the administrative software. 57% of the hospitals reported to implement a policy of MDRB monitoring. More than half patients considered to be at risk (eg, multiple hospitalizations, previous history of antibiotic treatment) was concerned in the majority of sectors welcoming patients with emerging MDRB. The most frequently screened MDRB are respectively MRSA (46%), carbapenemase-producing Enterobacteriaceae (44%), glycopeptide-resistant Enterococci (44%) and extended-spectrum beta-lactamase-producing Enterobacteriaceae (41%).

## Conclusion

The components of the MDRB control which are collected within the hospitals of the region have shown critical points, especially in the monitoring of patients with MDRB. Specific policy focusing MDRB should be reinforced in the region.

## Disclosure of interest

None declared

